# Robust and continuous oil/water separation with superhydrophobic glass microfiber membrane by vertical polymerization under harsh conditions

**DOI:** 10.1038/s41598-020-78271-9

**Published:** 2020-12-08

**Authors:** Seeun Woo, Hong Ryul Park, Jinyoung Park, Johan Yi, Woonbong Hwang

**Affiliations:** Department of Mechanical Engineering, Pohang University of Science and Technology (POSTECH), Pohang, Gyeongbuk 37673 Republic of Korea

**Keywords:** Materials science, Nanoscience and technology

## Abstract

We report a robust and continuous oil/water separation with nanostructured glass microfiber (GMF) membranes modified by oxygen plasma treatment and self-assembled monolayer coating with vertical polymerization. The modified GMF membrane had a nanostructured surface and showed excellent superhydrophobicity. With an appropriate membrane thickness, a high water intrusion pressure (< 62.7 kPa) was achieved for continuous pressure-driven separation of oil/water mixtures with high flux (< 4418 L h^−1^ m^−2^) and high oil purity (> 99%). Under simulated industrial conditions, the modified GMF membrane exhibited robust chemical stability against strong acidic/alkaline solutions and corrosive environments. The proposed superhydrophobic composite coating technique is simple, low cost, environmentally friendly, and suitable for the mass production of scalable three-dimensional surfaces. Moreover, its stability and customizable functionality offers considerable potential for a wide range of novel applications.

## Introduction

Numerous industrial activities involving petroleum, minerals, and pharmaceuticals generate copious amounts of emulsified wastewater, which pose a significant threat to ecosystems^1–3^. Because oil and water mixtures (a dispersion or emulsion) are multiphase systems, their separation is inherently an interfacial problem. Recently, considerable academic attention has been given to achieving effective oil/water separation using materials with special wettability to water and oil^4–10^. Superhydrophobic and superhydrophilic materials are effective agents for oil/water separation, and have enabled efficient and easy oil removal through selective oil filtration^11–13^ or absorption^14–17^. For example, Feng et al. first developed a coated mesh film simultaneously exhibiting superhydrophobicity and superoleophilicity that can effectively and rapidly separate oil/water mixtures via a simple and inexpensive process^18^. Meanwhile, Liang et al. fabricated a polyurethane sponge with excellent oil/water separation properties by modifying it with octadecyltrichlorosilane self-assemblies^19^. However, most reported metal mesh-type filters cannot be used to separate the micron-sized oil/water emulsions that are produced in industry, as their pores are larger than the oil droplets^20,21^. Absorption-type separators are easily contaminated and require frequent replacement. In addition, secondary treatment processes such as incineration produce environmental pollutants like carbon monoxide and sulfur oxides (SO_*x*_). This has a negative impact on cost, flux, and recyclability, and limits continuous separation^22,23^.

In recent years, to overcome these shortcomings, various polymeric, carbon nanotube, and glass fiber membranes have been studied, owing to their ease of operation and high separation efficiency. Yang et al. reported a facile hydrophilization method via the co-deposition of polydopamine and polyethyleneimine on a polypropylene microfiltration membrane for oil-in-water emulsion separation^24^. Saththasivam et al. achieved fast and efficient separation of emulsified oil/water mixtures by growing MnO_2_ nanorods on a carbon nanotube membrane^25^. However, polymeric membranes are generally weaker than their inorganic counterparts, and the high cost of carbon nanotube membranes limits their mass production, although they exhibit both high efficiency and mechanical strength^24–26^. Therefore, glass fiber membranes are considered the most suitable material for industrial oil/water separation, due to their high mechanical strength, low cost, chemical inertness to acids, and corrosion resistance^27,28^. Reacting the silanol groups that naturally occur on the glass surface with various organic compounds (particularly organosilanes) has been actively studied^29–32^. Although it has been widely demonstrated that superhydrophobicity can be imparted to glass materials, the fabrication of superhydrophobic glass fibers is still unsatisfactory in terms of cost efficiency, durability, and processability, which significantly hinders their practical applications. Most of the current reports include amino-silica particle synthesis and trimethoxymethylsilane reflux methods, which involve complex and time-consuming processes and cannot readily be adapted to produce large-scale or complex three-dimensional surfaces^33–35^.

In this paper, we developed a robust and continuous oil/water separation with nanostructured glass microfiber (GMF) membranes via vertical polymerization. In general, vertical polymerization of a molecule is considered to be a defect in the self-assembled monolayer (SAM) coating method^36,37^. Typical SAM coating is a horizontal molecular coating with low surface energy on the target surface^38^. However, through vertical polymerization, it is possible to significantly lower the surface energy, and also fabricate a surface with high roughness, thereby producing a superhydrophobic GMF membrane. The vertical polymerization coating method can be used for the mass production of large-scale and complex three-dimensional substrates. Moreover, the modified GMF membranes display high separation efficiency for various oil/water mixtures (> 99.0%), high water intrusion pressure (62.7 kPa) and high separation flux (4418 L h^−1 ^m^−2^). In particular, the separation flux of the modified GMF membrane is higher than the separation flux of the recently developed superhydrophobic filters, despite maintaining its superhydrophobicity^39,40^. The modified membranes could be used for continuous high-pressure oil/water separation while maintaining an extremely low water content of the filtrate (100.7 ppm at 500 mL), and were successful in separating oil/water mixtures with wide-ranging oil/water ratios. The coated surface exhibits an excellent chemical resistance compared to the wettability-modification literatures^41,42^, and does not exhibit any deterioration of wettability even in chemically severe environments, such as strongly acidic, alkaline, or saline conditions. The superhydrophobic nanostructured membranes capable of separating oil/water mixtures in these harsh environments have considerable potential for a wide range of novel applications.

## Results and discussion

### Superhydrophobic nanostructured GMF membrane

Low surface energy is a critical factor for obtaining superhydrophobicity and underwater superoleophobicity. This can be achieved by introducing a silane coupling agent (Supplementary Fig. S1). Hydroxyl (–OH) functional groups are produced on the surface of the pristine GMF substrate during the oxygen plasma treatment. The high surface energy of the hydroxyl functional groups implies that a small number of H_2_O molecules are attracted to the substrate surface^43^. Hydrolyzation with a silane coupling agent causes silanol groups to attach to the ends of octadecyltrichlorosilane (OTS) molecules. The formation of a self-assembled monolayer is postulated to occur through a condensation reaction between the hydroxylated substrate and hydrolyzed chlorine atoms of the OTS. The hydrolyzed OTS molecules physically adsorb onto the substrate via hydrogen bonding, ultimately forming Si_substrate_–O–Si_silane_ bonds and Si_silane_–O–Si_silane_ cross-linked covalent bonds^44^.

**Figure 1:**
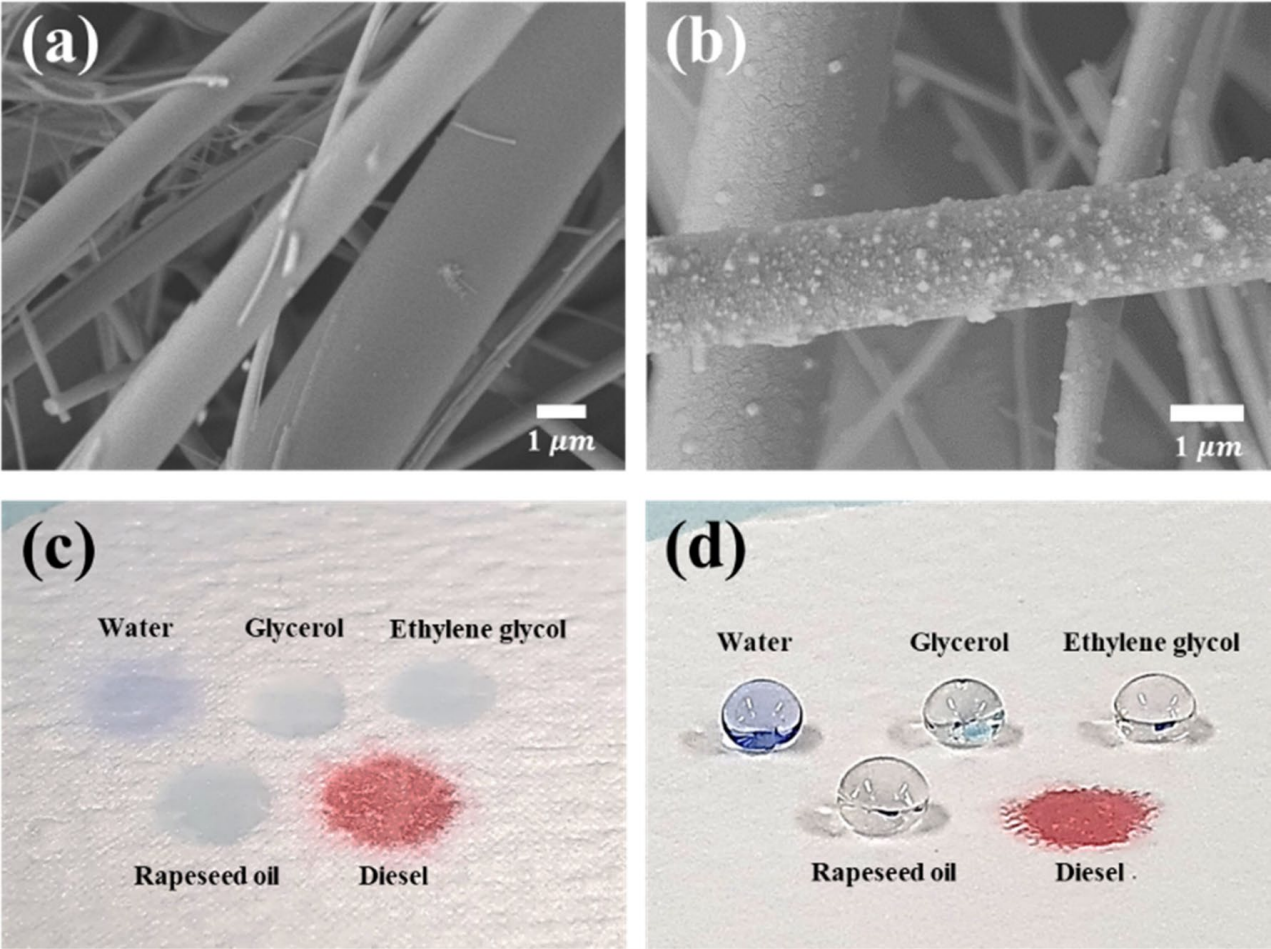
SEM images of (**a**) pristine GMF membrane and (**b**) modified GMF membrane. Optical images of liquid droplets with different surface tensions on (**c**) pristine GMF membrane and (**d**) modified GMF membrane.

The field-emission scanning electron microscopy (FE-SEM) images in Fig. 1a,b show the pristine and surface-treated GMF membranes. The GMF membrane exhibited distinct morphological changes after the surface treatment. These changes demonstrate the formation of nanostructures by vertical polymerization^45^. Vertical polymerization induces the formation of micro- or nano- structures depending on a sufficiently long reaction time or the degree of water solubility in solvents. The presence of water in the solution increases the probability of reaction between the OTS molecules and the formation of covalent bonds^46–49^. Under vertical polymerization conditions, the OTS molecules react simultaneously with the surface and other OTS molecules, resulting in a vertical structure on the surface. (Supplementary Fig. S2). Thus, vertical polymerization differs from self-assembled monolayer coating, which does not affect the surface morphology. Owing to morphological and chemical changes, the wettability of the surface was significantly different after surface treatment (Fig. 1c,d). Droplets of various liquids completely wet the surface of the pristine GMF membrane, showing superhydrophilicity, whereas the surface-treated GMF membrane exhibited non-wetting behavior; all the liquids (except diesel) formed nearly spherical droplets on the OTS-coated surface. These intrinsic wetting properties can be characterized as superoleophilic when the surface tension of the liquid droplet is less than 48 mN/m. The OTS-coated substrate exhibits an affinity for oils because the long carbon chains at the surface share structural similarities with the hydrocarbons in the oils^50^.

**Figure 2:**
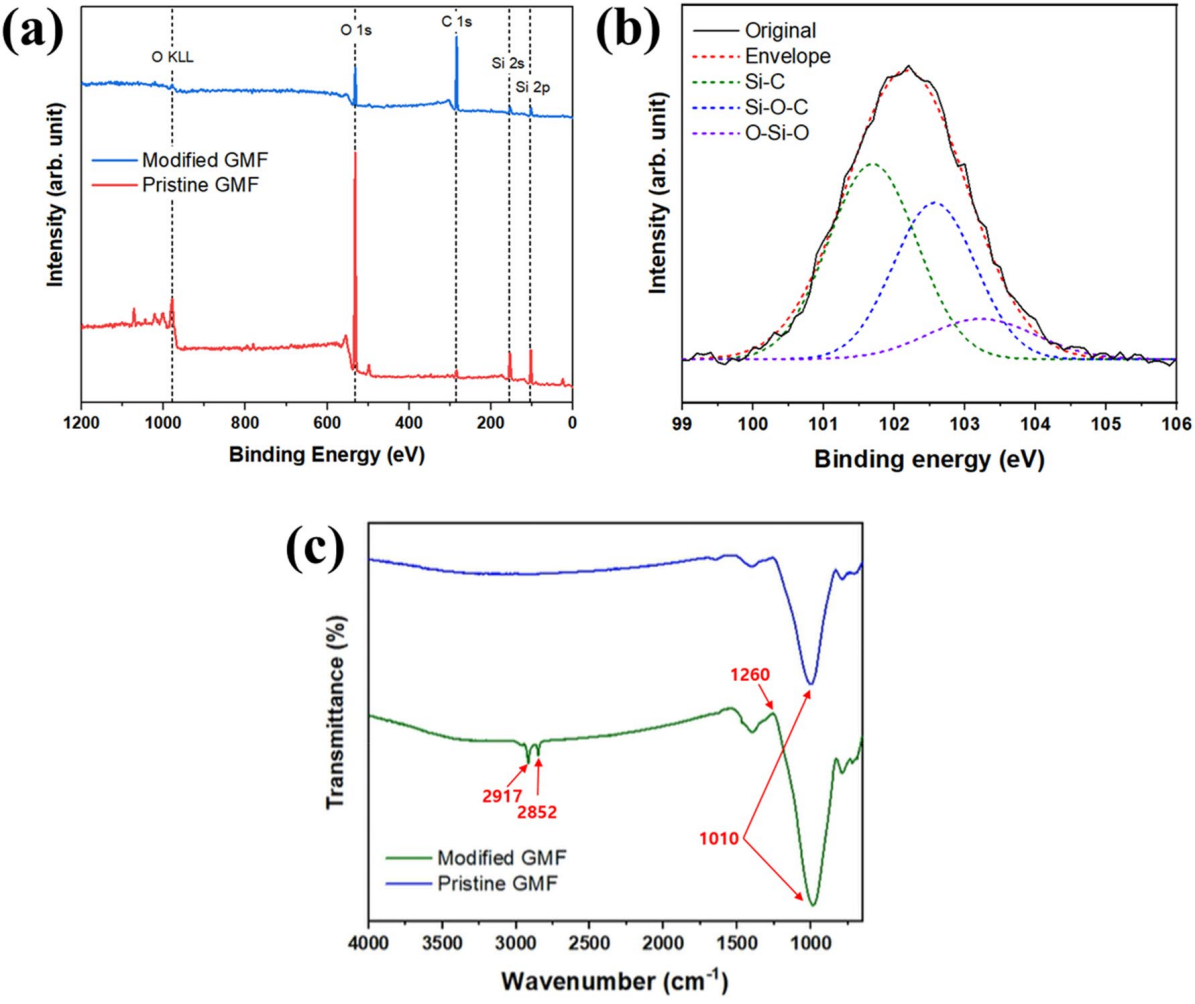
(**a**) Full high-resolution XPS spectra of pristine and modified GMF membranes. (**b**) Si 2p spectrum of modified GMF membrane. (**c**) FT-IR spectrum of pristine and modified GMF membranes.

X-ray photoelectron spectroscopy (XPS) and Fourier transform infrared spectroscopy (FT-IR) were performed to confirm whether there was adhesion between the GMF surface and the OTS coating layer. Figure 2a is an XPS spectrum showing that the original GMF membrane was composed of silicon and oxygen. The binding energies were measured to be 102.1 and 531.8 eV for the Si 2p and O 1 s peaks, respectively. After the coating process, the intensity of the C 1 s peak increased, consistent with the deposition of alkyl chains at the surface. The high-resolution Si 2p spectrum was used to investigate the chemical groups introduced onto the plasma-treated surface by the self-assembled monolayer coating process. The Si 2p spectrum (Fig. 2b) is composed of three strong peaks at 101.6, 102.3, and 103.1 eV, corresponding to Si–C, Si–O, and O–Si–O bonds, respectively. The concentration of each type of bond was 50.05%, 37.32%, and 12.63%, respectively. The organosilane self-assembled monolayer was successfully coated through S–O bonding via a condensation reaction between the hydrolyzed OTS and the hydroxylated surface^51,52^. As shown in Fig. 2c, FT-IR measurements in the 500–4000 cm^−1^ range and a baseline calibration were performed. The peak at 1010 cm^−1^ of both GMFs is the absorption peak of the Si–O–Si groups. In the spectrum of the coated GMF membrane, the peak for Si–C 1260 cm^−1^ is visible and the peaks at 2917 and 2852 cm^−1^ were attributed to the stretching vibration of aliphatic C–H groups. The observation of two slightly different frequencies indicates the successful grafting of the coating material onto the substrate of pristine GMF membrane.

**Figure 3:**
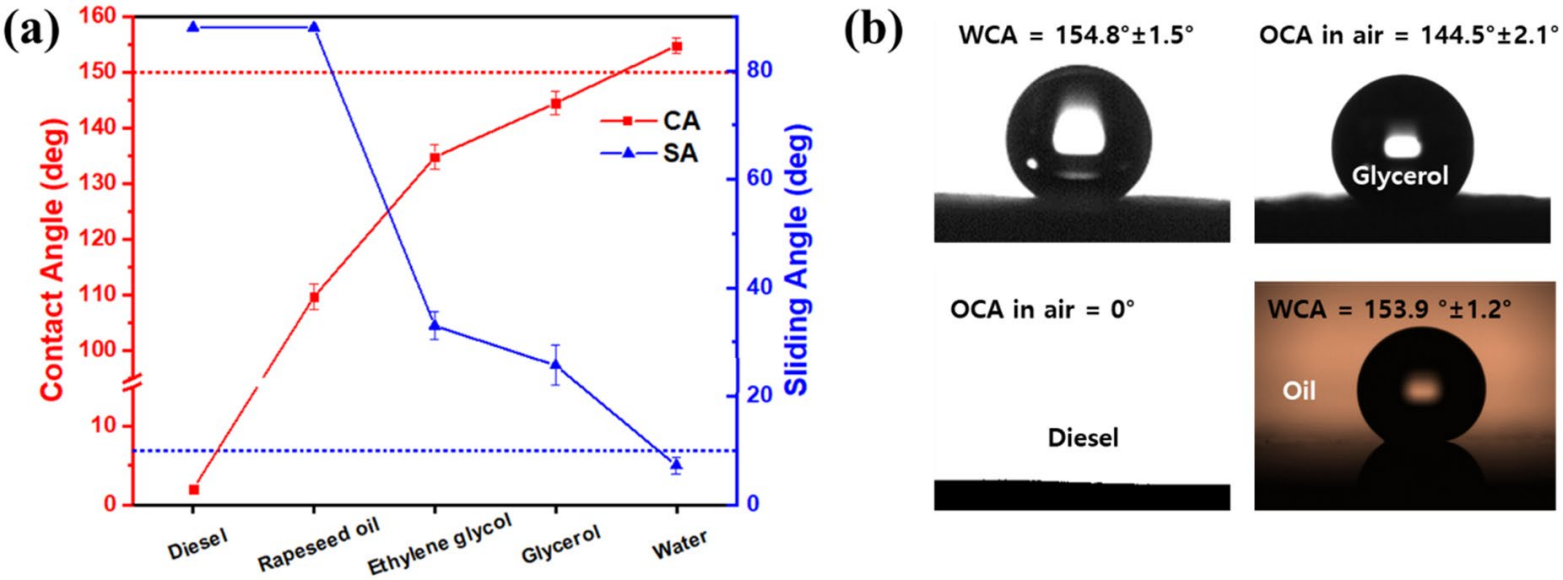
(**a**) CAs and SAs of liquid droplet in air as a function of surface tension. (**b**) Typical CA measurements of modified GMF membrane. *OCA* oil contact angle.

The wettability of the superhydrophobic GMF membrane was measured in air and under oil using liquids with different surface tensions (*γ*_*sl*_) (Fig. 3): DI water (*γ*_*sl*_ = 72.0 mN/m), glycerol (*γ*_*sl*_ = 64.0 mN/m), ethylene glycol (*γ*_*sl*_ = 47.3 mN/m), rapeseed oil (*γ*_*sl*_ = 33.8 mN/m), and diesel (*γ*_*sl*_ = 25.8 mN/m)^53,54^. In air, the surface exhibited superhydrophobic behavior toward water, with spherical water droplets having water contact angles (WCAs) of 154 ± 1.5° and sliding angles of 8 ± 0.5°. The water wetting behavior on the coated membrane was greatly enhanced compared with the original uncoated membrane, which had a water contact angle of 0°. This can be attributed to the synergistic effect of the hydrocarbon groups present in the OTS coating material and the nanoscale roughness of the fabricated membrane. With the use of glycerol, ethylene glycol and rapeseed oil, the contact angles were 144.5 ± 2.1°, 134.8 ± 2.3° and 109.7 ± 2.3°, respectively. When the diesel droplet contacted with the fabricated surface, it rapidly spread out and penetrated the membrane, with a contact angle of 0° above the surface. When the membrane was placed in diesel, the WCAs were of values 153.9 ± 1.2°. The trapped oil layer reduces the contact area of the surface–water interface, resulting in underoil-superhydrophobicity. Therefore, the wettability of the as-prepared GMF membrane indicated the possibility of separating water and oil with a surface tension of less than 25.8 mN/m. Water-jet and floating tests were performed to confirm superhydrophobicity/superoleophilicity with visual effects (Supplementary Fig. S3). The water-jet tests were conducted by spraying a water jet onto the membrane surface at an impact velocity of about 20 mL/s. Owing to the water repellent nature of the membrane, the water jet bounced quickly on impact, and left no trace on the membrane surface. The water was sprayed in one place for 20 min, and the water bounced continuously off the surface without any disturbance, demonstrating the mechanical strength of the coating. The floating tests were conducted with superydrophobic and pristine GMF membranes. The presence of air pockets at the superhydrophobic interface allowed the modified membrane to float on the water surface. The air pockets contribute to the superhydrophobicity of the GMF membrane since most of the area underneath the water is a liquid–air interface rather than a liquid–solid interface. When oil was added to the beaker, the superydrophobic GMF membrane sunk through the oil and remained suspended at the oil/water interface, while the pristine GMF sunk to the bottom.

### Oil/water separation under harsh conditions

**Figure 4:**
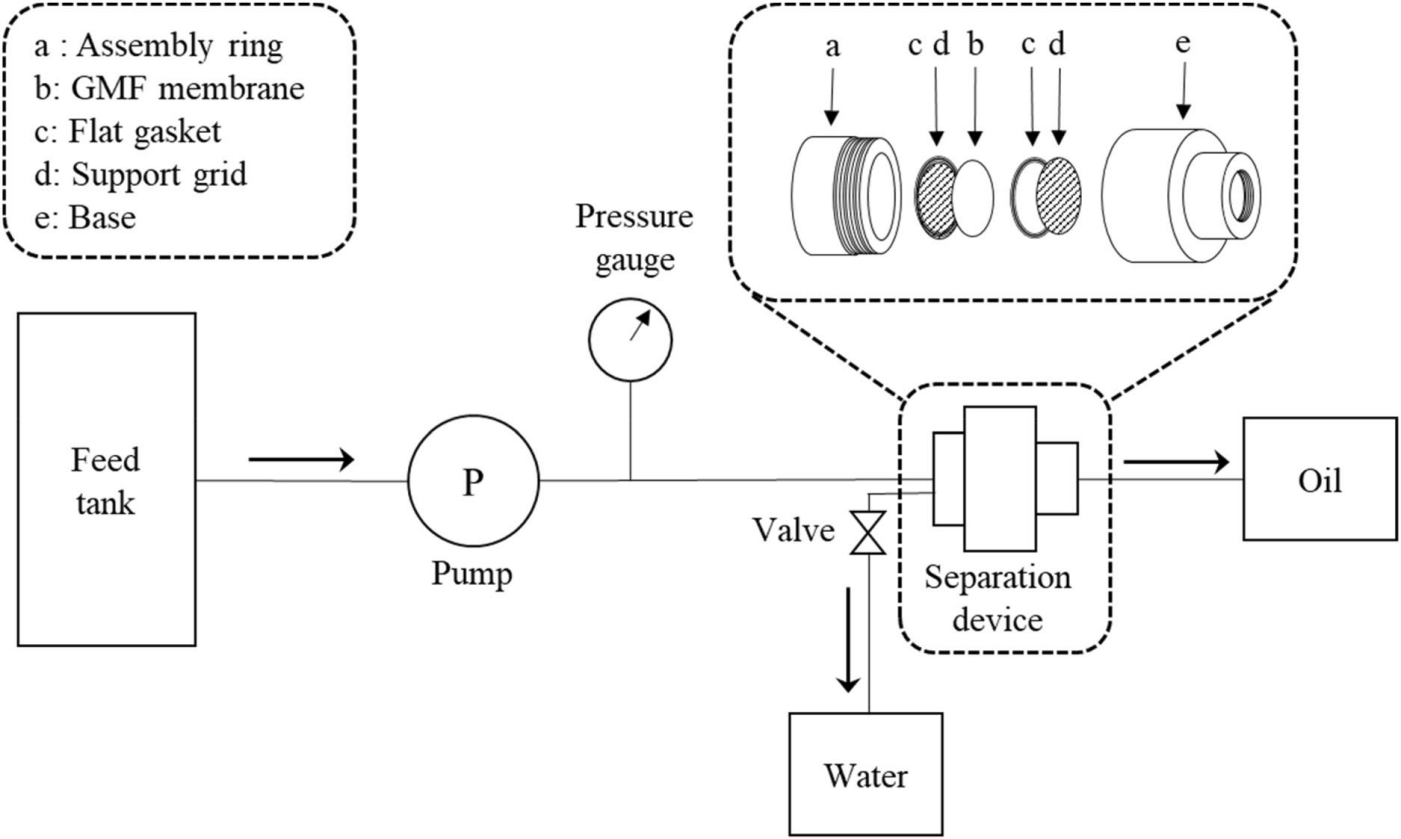
Schematic of the experimental set up for oil/water separation test.

To test the separation performance of membrane under different conditions, a pump system was used to produce pressure-driven flow of the oil/water mixture. Figure 4 schematically shows the experimental setup of the oil/water separation device. In the separation device, the GMF membrane is stacked between flat gaskets and support grids and secured between the assembly ring and base. The oil/water mixture was pumped from the feed tank to the separation device and a pressure gauge measured the pressure exerted by the mixture on the GMF membrane. The oil passes through the separation device while water is blocked by the membrane and discharged through the valve.

**Figure 5:**
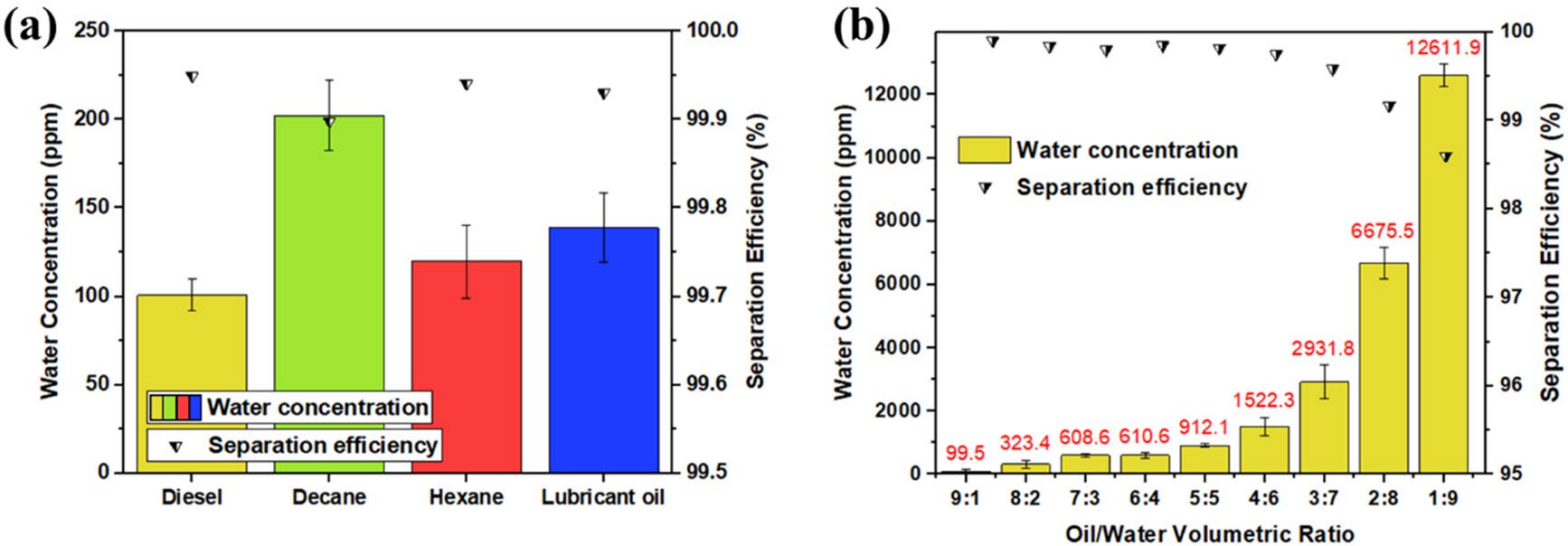
Water concentration of separated oil and separation efficiency of the superhydrophobic GMF membrane for (**a**) various light oil/water mixtures and (**b**) different volumetric ratios of oil/water mixtures.

Figure 5a displays the separation efficiency of the modified GMF membrane for different light oil/water mixtures (diesel, decane, hexane, and lubricant oil). High separation efficiency ranging from 99.95 to 99.90% was observed for each oil/water mixture, with the water content in the separated oil ranging from 100.7 to 202.2 ppm. Moreover, separation experiments were performed with a heavy oil/water mixture. Supplementary Fig. S4 shows the separation efficiency and water concentration. Because the water solubility and viscosity of these light and heavy oils are similar, there was a negligible difference in separation efficiency. To determine if the separation efficiency is dependent on the initial composition of the oil/water mixture, mixtures with oil/water with volumetric ratios ranging from 9:1 to 1:9 were fed at a constant pressure through the modified membrane (Fig. 5b). The separation efficiency decreased as the percentage of water in the mixture increased. We speculate that this could be related to the pressure exerted on the GMF membrane. While the mixture flows at the same flow rate, the water does not pass through the membrane; therefore, the cumulative pressure increases. The membrane cannot withstand this pressure, so water droplets gradually pass through.

**Figure 6:**
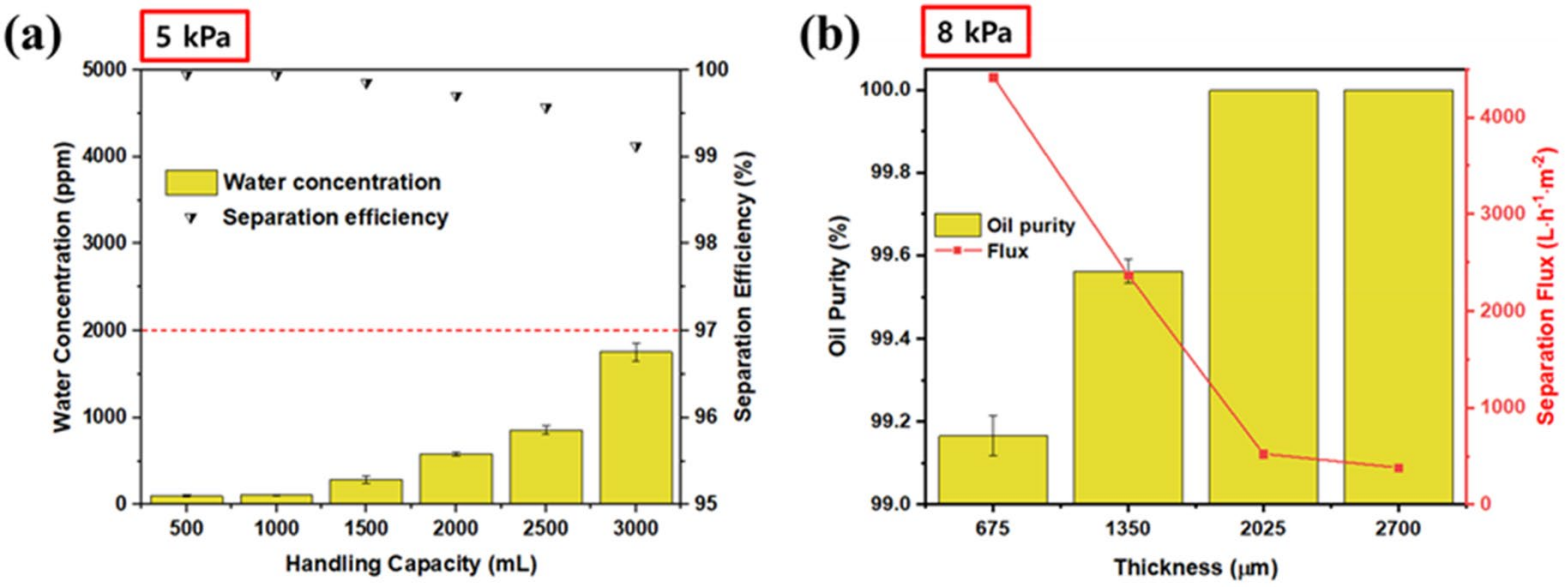
(**a**) Water concentration of separated oil and separation efficiency as a function of handling capacity for continuous separation. (**b**) Oil purity and separation flux of modified GMF membrane as a function of membrane thickness.

For the GMF membrane to be practical for oil/water separation, it should be able to withstand a certain pressure so that water cannot pass through it. The membrane detailed herein showed excellent pressure resistance. In addition, the water concentration of the filtered mixture retained a purity of over 99.8% throughout continuous oil/water separation. Figure 6a shows the water concentration of the filtered mixture for a continuous oil/water separation test (4:1 v/v diesel/water) up to the accumulation of 3000 mL. The pressure of the mixture on the membrane was constantly controlled to 5 kPa during the continuous separation. The water concentration in the separated oil remained below 1700 ppm, demonstrating that the GMF membrane could effectively separate 3000 mL of the oil/water mixture. The separation was conducted with a single small membrane with an effective surface area of 19.6 cm^2^. Considering the facile method of coating the membrane, much larger membranes could easily be produced; thus, there is a promising possibility of using this technique for industrial-scale oil/water separation applications. Figure 6b shows the separation flux of the modified GMF membranes with different thicknesses. The pressure of the mixture on the membrane was controlled at 8 kPa. The separation flux decreased from over 4418 to 382 L h^−1^ m^−2^ as the membrane thickness increased from 675 to 2700 μm, while the oil purity remained above 99.1%. The results are equivalent to, or higher than, previous studies of the separation flux on superhydrophobic surfaces, and are outstanding for industrial applications. It also showed a purity of more than 99%, like the previous oil–water separation literatures, despite the high separation flux^55,56^.

**Figure 7:**
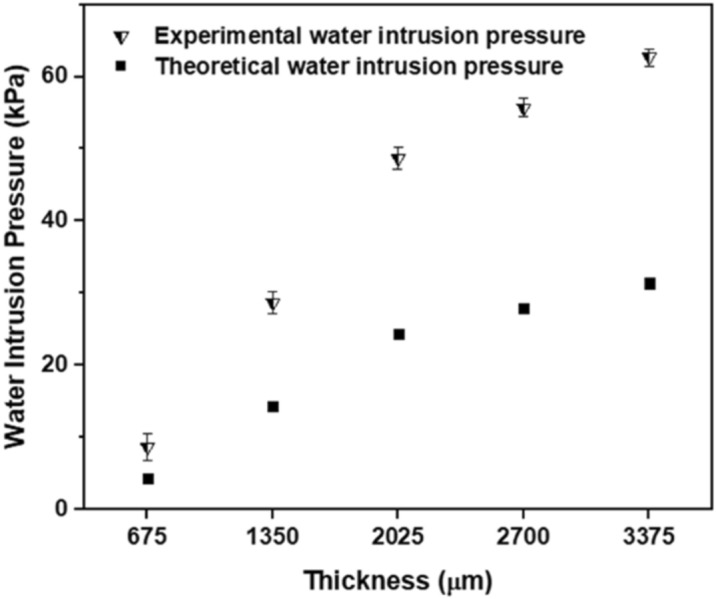
Experimental and theoretical water intrusion pressure of modified (superhydrophobic) GMF membrane.

The experimental water intrusion pressure was compared with the theoretical water intrusion pressure (Fig. 7). Thicker membranes have smaller pore diameters, which result in greater theoretical water intrusion pressure. The increase of water intrusion pressure with membrane thickness was true for both the experimental and theoretical cases. However, the experimental pressure was higher than the theoretical pressure for all thicknesses. This is because it is impossible to accurately determine the moment when the water droplet permeates through the membrane. In addition, as the membrane thickness increases, the inconsistency between the theoretical and experimental results increases, because the water droplets take a longer time to pass through the membrane and be noticed.

**Figure 8:**
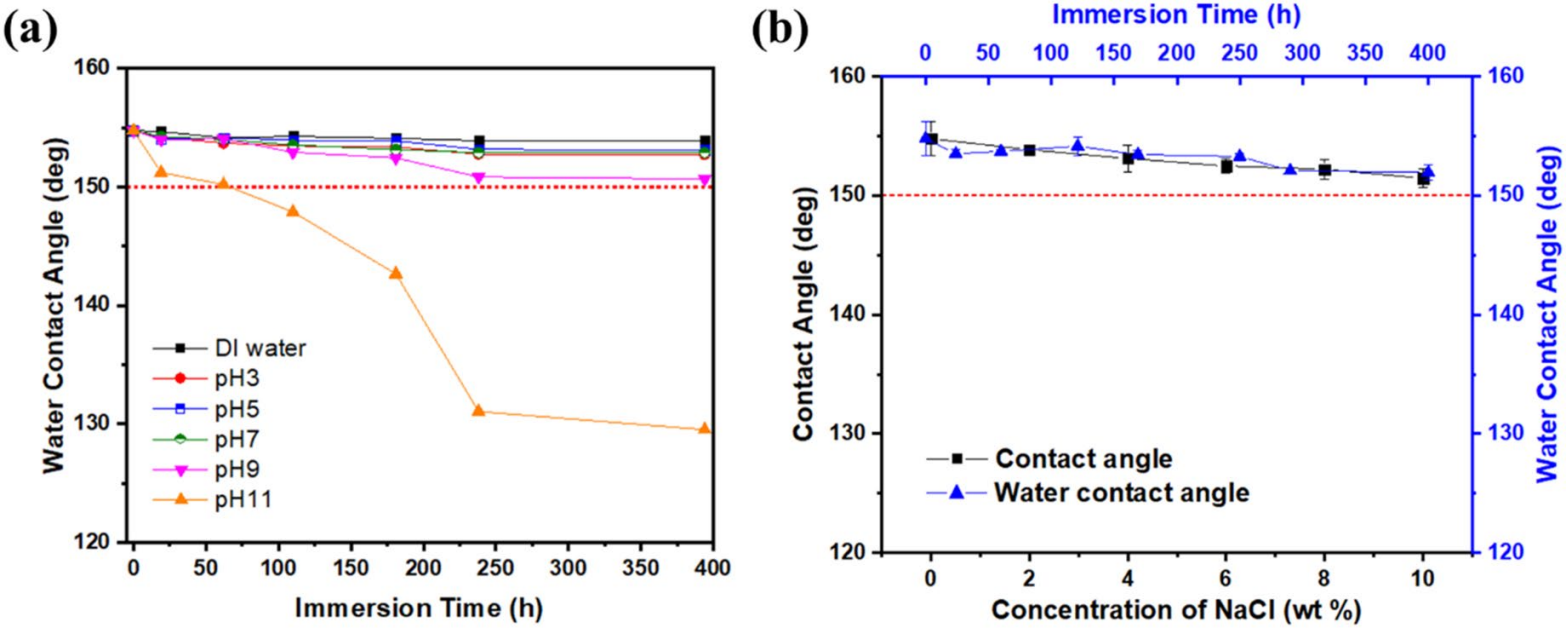
Influence of solutions with (**a**) different pH and (**b**) salinity on the wettability of modified (superhydrophobic) GMF membrane.

Outstanding anticorrosion properties are a necessity for achieving continuous oil/water separation in real industrial processes. The chemical stability of the modified GMF membrane was tested under extreme conditions and the surface damage was investigated by measuring the WCA. First, the superhydrophobic GMF membrane was immersed in acidic and alkaline corrosive solutions with a wide pH range from 3–11. As shown in Fig. 8a, the superhydrophobic GMF membrane demonstrated excellent long-term stability with a WCA of 151° when immersed for 400 h in solutions with a pH of ≤ 9. However, after approximately 75 h in a solution with pH 11, the WCA reduced below 150°, resulting in the loss of superhydrophobic properties. After 400 h at the higher pH, the WCA had decreased below 130°, showing only conventional hydrophobicity. Thus, the membrane has relatively lower chemical durability in highly alkaline solutions than in acidic solutions. The stability of the modified membrane in saline environments was examined via saline solution contact angle and durability tests in 1 M NaCl solution, as shown in Fig. 8b. The membrane effectively repelled droplets of 0–10 wt% NaCl, and the WCA remained above 150° after immersion in 1 M NaCl aqueous solution for 400 h. These results indicate that the coated composite membrane is particularly corrosion resistant under the severe conditions established by the experiment.

Also, flame-retardant properties can significantly expand the scope of application and lifetime of the GMF membrane and ensure the durability of its surface properties since ordinary glass fibers can burn quickly. Horizontal combustion experiments were carried out on the pristine and modified GMF membranes, as shown in Supplementary Fig. S5. The uncoated GMF ignited vigorously within 1 s when exposed to direct flame. The surface exposed to the flame was completely burned out within 4 s, without leaving any residue. In contrast, the superhydrophobic GMF membrane exhibited flame-retardant properties, with the flame spreading less vigorously. The modified GMF membrane was totally burned out after 13 s when exposed to direct flame. Thus, it may possibly be used as a flame-retardant membrane with potential for reducing the risk of fire.

## Conclusion

In summary, we developed a vertical polymerization coating method for producing superhydrophobic nanostructured GMF membranes. We studied the robustness of this material for pressure-driven oil/water separation and its durability even in harsh conditions. The designed GMF membrane displayed superb superhydrophobicity and successfully separated various oil/water mixtures with separation efficiencies exceeding 99.9%. As the proportion of water increased in the oil/water mixture, the separation efficiency was reduced by the cumulative pressure during continuous separation, but still showed a separation efficiency of 98.5% or more. The membrane exhibited good durability at high driving pressures, while the water content in the filtrate remained extremely low (100.7 ppm at 500 mL). The enhanced intrusion pressure maintained an oil purity of 99.9% even when the driving pressure of oil/water separation reaches 8 kPa. When used under severe conditions, such as strong acids, alkaline aqueous solutions, or saline environments, the as-obtained membrane could maintain its wettability. As a result, our proposed vertical polymerization coating method is suitable for the mass production of large-scale three-dimensional superhydrophobic surfaces. Moreover, it is believed that the ideal separation stability against applied pressure and chemically harsh environments have considerable potential for a wide range of novel applications.

## Methods

### Surface fabrication and characterization

GMF membranes (Grade GF/B, diameter: 5 cm, weight: 143 g/m^2^) were supplied by Whatman (UK), *n*-hexane (> 96% purity) was purchased from Samchun Chemicals (Republic of Korea), and octadecyltrichlorosilane (OTS) was obtained from Sigma-Aldrich (USA). The process for fabricating the superhydrophobic GMF membrane is depicted in supplementary Fig. S6. The GMF membrane was oxygen plasma-treated using a plasma system (CUTE, Femto Science, Republic of Korea). The internal pressure and discharge power of the reactor were 60 mTorr and 100 W, respectively; the treatment time was 1 min; and the oxygen supply rate was set to 20 sccm to promote hydroxylation of the surface. The superhydrophobic coating process was conducted by organosilane self-assembled monolayer coating the hydroxylated surface in a mixture of n-hexane and OTS (1000:1 v/v) for 30 min followed by oven drying at 60 °C for over 10 min. HDFS coating, well known as self-assembled monolayer coating, was attempted in the same way as OTS, and lowers surface energy by fluorinating the hydroxide surface. However, it is not suitable for oil/water separation because it produces an oleophobic surface that also repels water. The fabricated superhydrophobic/superoleophilic GMF membrane was evaluated by measuring the contact angle (CA) and sliding angle (SA) of 5 μL droplets of various liquids with a CA analysis device (SmartDrop, FEMTOFAB, Republic of Korea). Average CA and SA values were obtained based on the five measurements from different locations across the membrane surface. High-resolution field-emission scanning electron microscopy (FE-SEM; JSM-7401F, JEOL, Japan) was used to observe the surface morphology of the GMF membrane. X-ray photoelectron spectroscopy (XPS, ESCALAB 250XI, Thermo Fisher Scientific) and Fourier transform infrared spectroscopy (FT-IR, FT-4100, JASCO) were employed to analyze the chemical state of the surface^57^.

### Oil/water separation experiment setup

Oil/water mixtures (4:1 v/v) were prepared by mixing 400 mL of oil (diesel, decane, hexane, or lubricant oil) with 100 mL of water. The as-prepared GMF membrane was mounted on the filtering apparatus (Supplementary Fig. S6) and wetted with the corresponding oil. For each test, 500 mL of the oil/water mixture was pumped into the filtration apparatus for separation. After oil/water separation, the trace water content of the separated oil was measured using a compact Karl Fischer coulometer (C20, Mettler/Toledo, Switzerland). The separation efficiency (*η*, %) was calculated using Eq. (1)^58,59^.

1 $$ \eta { } = \left( {1 - \frac{{C_{1} }}{{C_{0} }}} \right) \times 100\% , $$

where *C*_1_ and *C*_0_ are the contents of the liquid phases in the mixtures before and after separation, respectively. The flux (*F,* L h^−1^ m^−2^) of the separation was calculated using Eq. (2)^60^.

2 $$ F = \frac{V}{TA}, $$

where *ν* is the volume of liquid that permeates through the as-prepared GMF membrane (h), and *A* is the effective separation contact area (m^2^). The theoretical water intrusion pressure (*P*_theo_, kPa) was calculated using Eq. (3)^61^.

3 $$P_{{{\text{theo}}}} = \, 2\gamma_{l1/2} \cos \vartheta /d, $$

where *γ*_*l*1*l*2_ is the interfacial tension between the water and oil (mN/m), *ϑ* is the water contact angle (WCA, °), and *d* represents the pore diameter of the membranes (m). All experimental data were determined as the average of five measurements.

## Supplementary Information

Supplementary Information.

## Data Availability

*Scientific Reports *requires a Data Availability Statement to be included in all submitted manuscripts (at the end of the main text, before the References section); see https://www.nature.com/srep/journal-policies/editorial-policies#availability.
