# Supplementary material for: Robust and continuous oil/water separation with superhydrophobic glass microfiber membrane by vertical polymerization under harsh conditions
Source: Sci Rep. 2020 Dec 8;10:21413. doi: 10.1038/s41598-020-78271-9 (PMC7722867; doi:10.1038/s41598-020-78271-9)
Supplement: Supplementary file 1 — Supplementary Information. [file 41598_2020_78271_MOESM1_ESM.docx]

**Supplementary Information**

**Robust and continuous oil/water separation with superhydrophobic glass microfiber membrane by vertical polymerization under harsh conditions**

Seeun Woo, Hong Ryul Park, Jinyoung Park, Johan Yi, and Woonbong Hwang


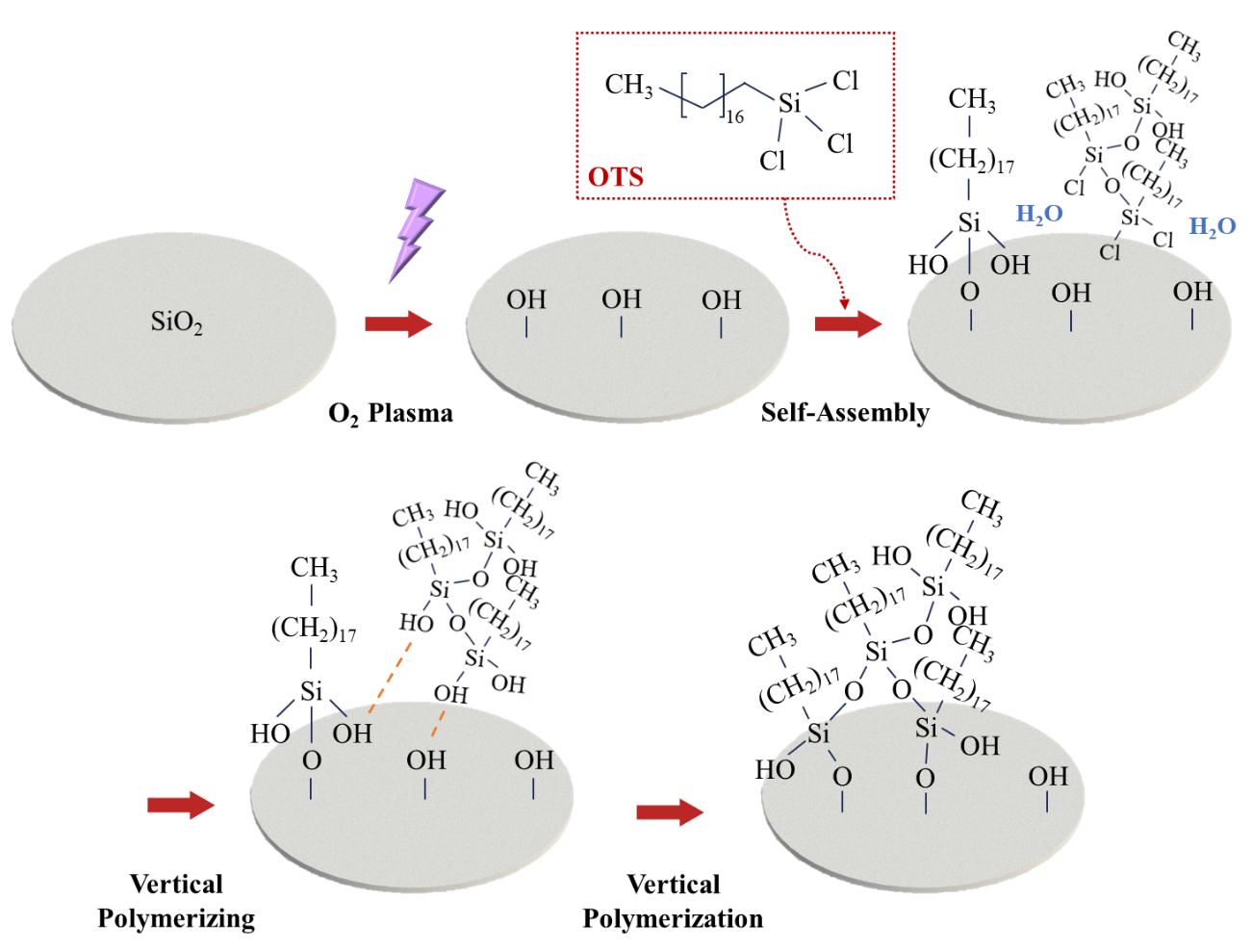


**Fig. S1.** Formation process of superhydrophobic GMF membrane.


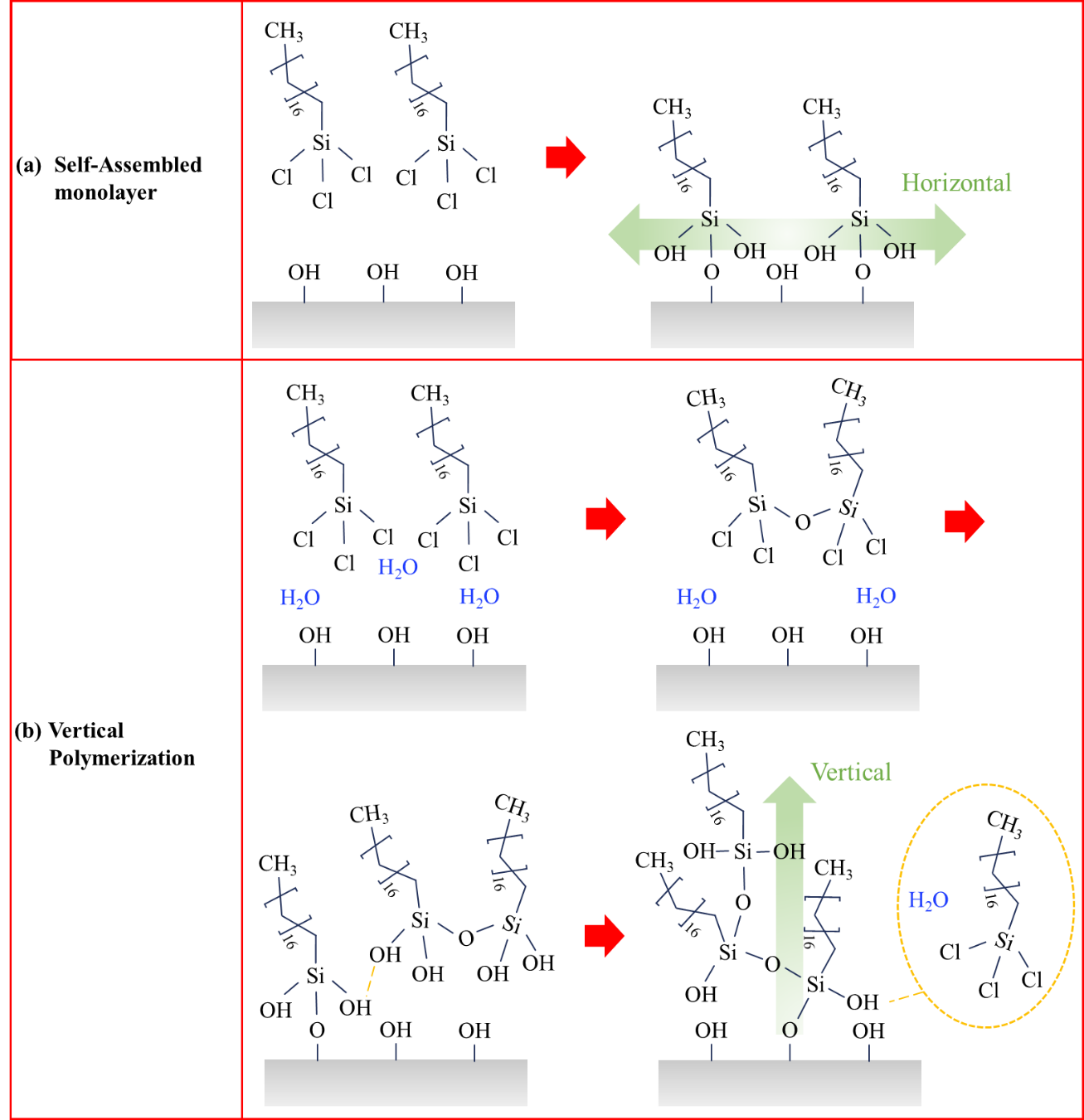


**Fig. S2.** Schematic diagram of (a) self-assembled monolayer and (b) the vertical polymerization mechanism of OTS molecules.


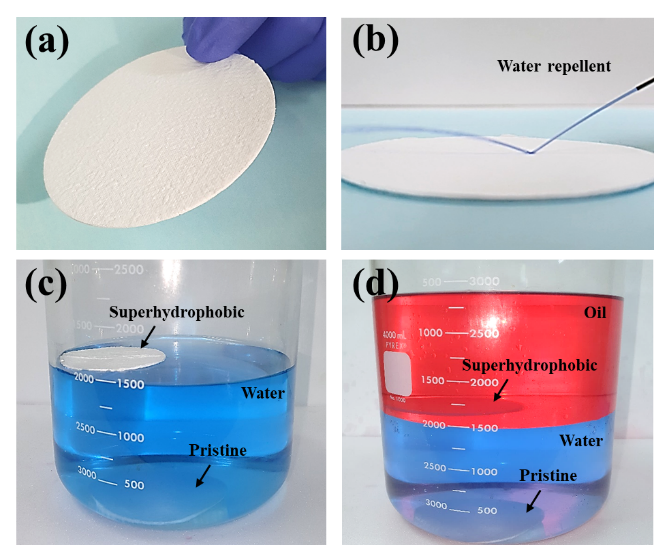


**Fig. S3.** Photographs of (a) surface-treated GMF membrane and (b) water-jet test on surface-treated membrane. Flotation of modified (superhydrophobic) and pristine GMF membranes in (c) water and (d) layered water/oil.


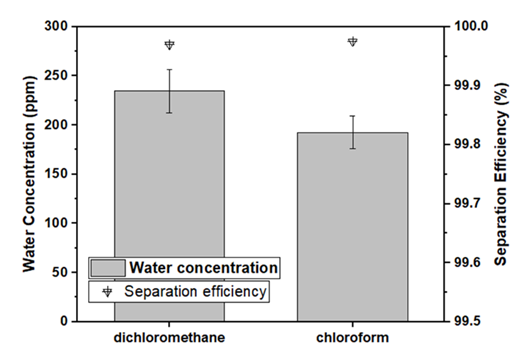


**Fig. S4.** Water concentration of separated oil and separation efficiency of the superhydrophobic GMF membrane for heavy oil/water mixtures


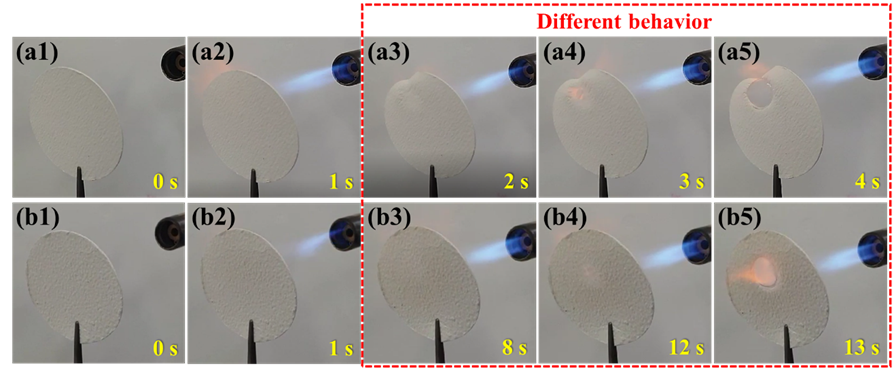


**Fig. S5.** Horizontal combustion tests of (a) pristine and (b) modified (superhydrophobic) GMF membranes.


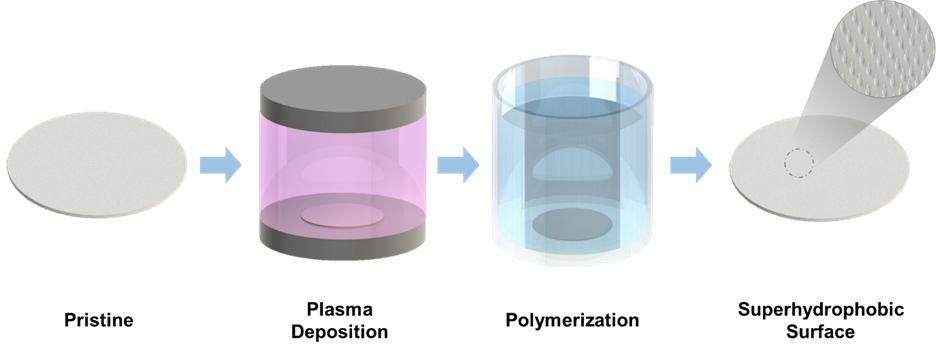


**Fig. S6.** Schematic illustration of the nanostructured GMF membrane fabrication process.


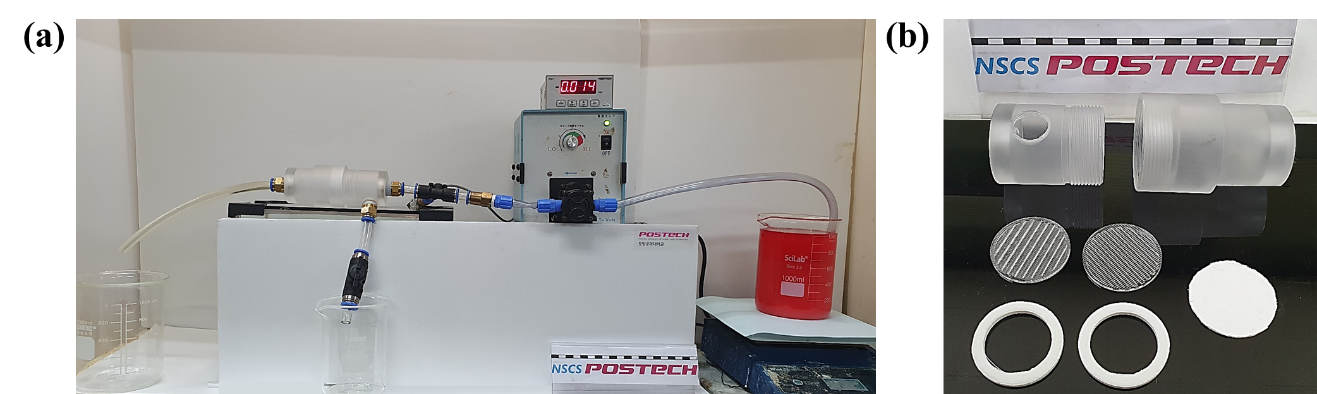


**Fig. S7.** (a) Continuous oil/water separation device and (b) assembly structure.
